# All-cause and cause-specific mortality rates for Kisumu County: a comparison with Kenya, low-and middle-income countries

**DOI:** 10.1186/s12889-022-14141-5

**Published:** 2022-09-27

**Authors:** Wanjiru Waruiru, Violet Oramisi, Alex Sila, Dickens Onyango, Anthony Waruru, Mary N. Mwangome, Peter W. Young, Sheru Muuo, Lilly M. Nyagah, John Ollongo, Catherine Ngugi, George W. Rutherford

**Affiliations:** 1 Institute for Global Health Sciences, University of California, San-Francisco, USA; 2 Ministry of Health, National AIDS and STI Control Program (NASCOP), Nairobi, Kenya; 3 United Nations Poulation Fund, Vientiane, Laos; 4 Kisumu County Department of Health, Kisumu, Kenya; 5 US Centers for Disease Control and Prevention (CDC), Division of Global HIV & TB, Nairobi, Kenya; 6 Global Programs for Research and Training (GPRT), Nairobi, Kenya; 7 Ministry of Health, Office of Director General, Nairobi, Kenya; 8 Jaramogi Oginga Odinga Teaching and Referral Hospital, Kisumu, Kenya

**Keywords:** Mortality, Cause of death, Kisumu, Kenya, LMICs

## Abstract

**Background:**

Understanding the magnitude and causes of mortality at national and sub-national levels for countries is critical in facilitating evidence-based prioritization of public health response. We provide comparable cause of death data from Kisumu County, a high HIV and malaria-endemic county in Kenya, and compared them with Kenya and low-and-middle income countries (LMICs).

**Methods:**

We analyzed data from a mortuary-based study at two of the largest hospital mortuaries in Kisumu. Mortality data through 2019 for Kenya and all LMICs were downloaded from the Global Health Data Exchange. We provided age-standardized rates for comparisons of all-cause and cause-specific mortality rates, and distribution of deaths by demographics and Global Burden of Disease (GBD) classifications.

**Results:**

The all-cause age-standardized mortality rate (SMR) was significantly higher in Kisumu compared to Kenya and LMICs (1118 vs. 659 vs. 547 per 100,000 population, respectively). Among women, the all-cause SMR in Kisumu was almost twice that of Kenya and double the LMICs rate (1150 vs. 606 vs. 518 per 100,000 population respectively). Among men, the all-cause SMR in Kisumu was approximately one and a half times higher than in Kenya and nearly double that of LMICs (1089 vs. 713 vs. 574 per 100,000 population). In Kisumu and LMICs non-communicable diseases accounted for most (48.0 and 58.1% respectively) deaths, while in Kenya infectious diseases accounted for the majority (49.9%) of deaths. From age 10, mortality rates increased with age across all geographies. The age-specific mortality rate among those under 1 in Kisumu was nearly twice that of Kenya and LMICs (6058 vs. 3157 and 3485 per 100,000 population, respectively). Mortality from injuries among men was at least one and half times that of women in all geographies.

**Conclusion:**

There is a notable difference in the patterns of mortality rates across the three geographical areas. The double burden of mortality from GBD Group I and Group II diseases with high infant mortality in Kisumu can guide prioritization of public health interventions in the county. This study demonstrates the importance of establishing reliable vital registry systems at sub-national levels as the mortality dynamics and trends are not homogeneous.

## Introduction

Countries are in constant need of reliable mortality and cause of death statistics to monitor population health and effectively respond with appropriate public health interventions [1, 2]. In general, high-income countries have mature vital and civil registration systems that can reliably estimate both overall mortality and cause-specific mortality rates [3]. In low-and middle-income countries (LMICs), however, vital registration systems are generally not well established, and documentation of mortality and its causes are poor [4, 5], resulting in uncertainty in estimated mortality rates [6, 7].

To help address this gap, some sub-Saharan African (SSA) countries have set up demographic surveillance sites (DSS) to provide cause-specific mortality rates by collecting cause of death (COD) data through verbal autopsies based on a standard verbal autopsy questionnaire [8–11]. Although an efficient way to provide accurate age and cause-specific mortality rates, the data are limited to the geographic areas covered by the DSS. Cause of death data from verbal autopsies have their limitations including recall bias and classification errors [11, 12]. Additionally, some countries in SSA, such as Kenya, have also relied on statistical models to provide mortality estimates [13]. Such models, however, are limited by the quality of the data provided and the differences in methods for ascertaining cause of death [13, 14]. Outside of routine comprehensive civil and vital statistic registries, some SSA countries have used direct methods that can mitigate some of these limitations by collecting primary mortality data from hospitals [15, 16] or mortuaries [17–20] to calculate mortality rates.

On a global level, the Institute for Health Metrics and Evaluation (IHME), an independent population health research center in Seattle, Washington, USA, provides rigorous and comparable measurement of the world’s most important health problems on its Global Health Data Exchange [21]. The exchange is the world’s most comprehensive global catalogue of data from vital registration systems, sample registration systems, household surveys (complete birth histories, summary birth histories, sibling histories), censuses (summary birth histories, household deaths) and DSS sites. The Exchange’s data catalogue is used to model and generate Global Burden of Disease (GBD) estimates on key health indicators including mortality.

Because these diverse methods for deriving mortality rates exist, mapping of mortality data onto the International Statistical Classification for Diseases and Health Related Problems 10th Revision, (ICD-10) coding system [22] and subsequently summarizing them according to the categories of the Global Burden of Disease (GBD) project [23] allow for comparison of data across different geographical areas. This paper aims to i) analyze all-cause and cause-specific mortality rates from a mortuary-based survey in Kisumu; ii) compare Kisumu mortality rates with mortality data for overall Kenya; and iii) compare Kisumu mortality data with mortality rates for LMICs (data from the Global Health Data Exchange).

## Methods

### Study design and population

We conducted a study in Kisumu County, which is located in the western part of Kenya and has a population of approximately 1.1 million people [24]. The study occurred at two of the county’s largest mortuaries, Jaramogi Oginga Odinga Teaching and Referral Hospital (JOOTRH) and Kisumu County Referral Hospital (KCRH). These two hospital-based mortuaries accounted for 42% of the deaths reported to Kisumu East City Registry, which received three quarters of all deaths in the county in 2017 [25]. We consecutively enrolled all decedents irrespective of age who had died at the hospitals or were brought in dead and admitted to the two mortuaries between April and July 2019. We abstracted demographic and cause of death data for decedents from mortuary records, hospital files and post-mortem reports. These data were used for re-certification and coding of cause of death using International Classification of Diseases and Related Health Problems 10th Revision (ICD10) codes. Comparative data for mortality in Kenya and LMICs were derived from the IHME’s Global Health Data Exchange. The exchange contains annual results through 2019 for all-cause and cause-specific deaths, and these are freely available online [21]. These results are available by country as well as by country or regional grouping using the socio-demographic index (SDI); a metric for measuring development; which has been found to be similar in LMICs [26]. A total of 90 countries have been grouped in the LMICs grouping in the data exchange; majority which are in SSA [21].

### Coding for cause of death

For Kisumu data, a panel of medical officers trained in cause-of-death determination and ICD10 coding abstracted data from hospital records onto a paper-based customized data collection tool that were used to document information to support the assignment of immediate, antecedent and underlying causes of death for each of the decedents. The abstracted data included a summary of conditions, medical history and diagnosis before death, presenting complaints, laboratory and radiological investigations, HIV status and symptoms documentation. An individual panel member assigned the final underlying cause of death on the tool, and a panel discussion was held to determine cause of death when it was not clear to individual panelists. Health record information officers assigned ICD10 codes based on the final cause of death, entered these data onto an electronic tool and submitted the codes to the study database.

Cause of death for Kisumu was analyzed using Analyzing Mortality Levels and Causes-of-Death (ANACoD) tool, version 2.0 (World Health Organization, Geneva, Switzerland). The Microsoft® Excel-based tool provided a stepwise approach that enabled the comprehensive analysis of ICD-10-coded data and categorized the underlying causes of death into GBD Groups I, II or III [23]. Group I causes include communicable diseases, maternal and perinatal conditions and nutritional deficiencies, Group II includes non-communicable diseases, and Group III includes external causes of mortality or injuries.

### Calculating mortality rates

For Kisumu County mortality rates, we first annualized the number of deaths for Kisumu County based on the proportion of deaths reported to the Kisumu East Civil Registry (in whose catchment area the participating mortuaries are located) during a 12-month period in 2019 and the reported coverage of all deaths in the county by the Kisumu East Civil Registry. We calculated the annual crude mortality rate by dividing the total number of deaths in 2019 by the reported population in the census conducted in the same year [24] and expressed it as deaths per 100,000 population. Age-specific and cause-specific mortality rates were calculated by dividing the numbers of deaths in an age group and the deaths assigned to each cause by the population in each category for that year and expressed as deaths per 100,000 population. To allow for comparison across the three geographical areas, we calculated age-standardized mortality rates (SMR) for all-cause mortality and GBD classes. This was done by applying the age-specific rates to the 2019 Kenya population distribution as the standard [24]. Decedents missing a cause of death, age or sex were not included in the analysis. For the Kenya and LMICs mortality rates per 100,000 population, the Global Health Data Exchange provides downloadable data tables on global all-cause and cause-specific deaths by geographical locations, GBD class, age, sex and year of interest. Summary tables were created to compare all-cause and cause-specific mortality data from Kisumu County, Kenya and LMICs by age, sex and GBD class.

## Results

### Kisumu study enrollment

In total, 1004 decedents were admitted into the two mortuaries during the study period; half of whom were male (50.9%), and the majority (69.4%) from JOOTRH. Of all the decedents, 66 (6.6%) were stillbirths and four (0.4%) had missing age. We analyzed data for these 934 decedents excluding stillbirths and those with missing ages for all-cause mortality.

From the 934, after excluding decedents who were either ineligible or unavailable (deteriorated, dead> = 48 hours, burns or already embalmed) for the study, there were 851 eligible decedents; 555 (65.2%) died at the hospital, and the remainder were brought in dead. Of the 555, we were able to retrieve hospital records for 456 (82.2%), and among these, 14 (3.1%) did not receive a final cause of death as their hospital records were incomplete. We analyzed data for 442 records that had a documented cause of death for cause-specific mortality.

### All-cause mortality

The all-cause SMR in Kisumu County was one and half times that of all of Kenya (1118 vs. 659 per 100,000 population respectively), and twice that of LMICs (547 per 100,000 population) (Table 1). Among women, the all-cause SMR in Kisumu (1150 per 100,000 population) was almost twice that of Kenya and double of LMICs (606 and 518 per 100,000 population, respectively). Among men, all-cause SMR in Kisumu (1089 per 100,000 population) was one and a half times higher than in Kenya (713 per 100,000 population) and nearly twice that of LMICs (574 per 100,000 population).

**Table 1:** Age-standardized all-cause mortality per 100,000 population by GBD class and sex; Kisumu, Kenya & LMIC 2019

	Kisumu			Kenya			LMIC		
	Both Sexes (column%)	Female (column%)	Male (column%)	Both Sexes (column%)	Female (column%)	Male (column%)	Both Sexes (column%)	Female (column%)	Male (column%)
Group I^a^	524 (46.8)	530 (46.1)	549 (50.4)	329 (49.9)	310 (51.1)	349 (49.0)	175 (32.0)	177 (34.1)	174 (30.3)
Group II^b^	537 (48.0)	595 (51.8)	448 (41.1)	286 (43.4)	272 (44.9)	300 (42.0)	318 (58.1)	304 (58.8)	329 (57.4)
Group III^c^	58 (5.2)	24 (2.1)	92 (8.5)	44 (6.7)	24 (4.0)	64 (9.0)	54 (9.8)	37 (7.1)	71 (12.3)
smr	1118 (100)	1150 (100)	1089 (100)	659 (100)	606 (100)	713 (100)	547 (100)	518 (100)	574 (100)

*SMR* standardized mortality rate, *LMIC* low- and middle-income countries

^a^Group I - Communicable, perinatal, maternal and nutritional conditions

^b^Group II - Non-communicable diseases

^c^Group III –Injuries

In Kisumu, communicable and non-communicable diseases contributed equally to all-cause SMR: 524 and 537 per 100,000 population, respectively (Table 1). Non-communicable diseases contributed most to all-cause SMR among women (51.8% of deaths or 595 per 100,000 population) while communicable diseases accounted for most deaths among men (50.4% of all deaths or 549 per 100,000 population). Injuries resulted in a greater percentage of deaths among men (8.5%) than women (2.1%) in Kisumu.

In Kenya, communicable diseases accounted for half of all-cause mortality (329 per 100,000 population), or 49.9% of all deaths. For both women and men in Kenya, communicable diseases contributed most to all-cause SMR (310 and 349 per 100,000 population respectively), representing 51.1 and 49.0% of deaths in the two groups. In LMICs, non-communicable diseases accounted for majority of all-cause SMR overall (58.1%), and by individual sex. Overall, Kenya and LMICs had higher all-cause mortality among men compared to women. Injury-related deaths affected more men than women in all geographical areas, with mortality from injuries among men at least twice that of women except in LMICs.

**Fig. 1:**
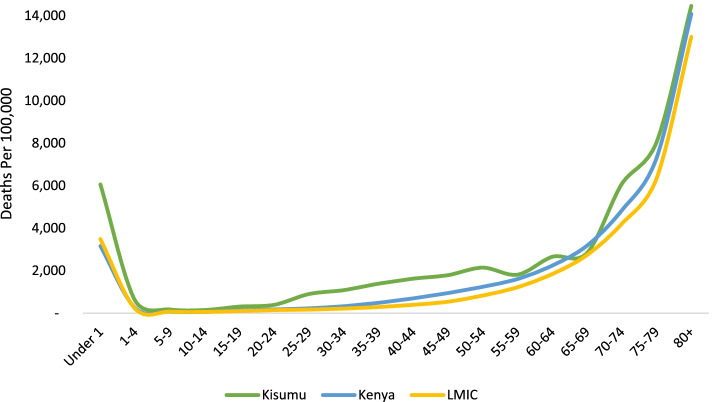
All-cause mortality by age in Kisumu, Kenya & LMICs, 2019

In Kisumu, the age-specific mortality rate among those under 1 (6058 per 100,000 population) was nearly twice that of Kenya and LMICs (3157 and 3485 per 100,000 population, respectively) (Table 2). Mortality rates tended to increase with age across all geographies after childhood, with Kisumu having higher mortality rates compared to Kenya and LMICs (Table 2 & Fig. 1).

**Table 2:** All -cause age-specific mortality rates per 100,000 population, Kisumu, Kenya & LMIC **2019**

Age (Years)	KISUMU MR (95% CI)			KENYA MR (95% CI)			LMIC MR (95% CI)		
		Female	Male	Both Sexes	Female	Male	Both Sexes	Female	Male
Under 1	6058 (5771–6355)	5130 (4759–5523)	6980 (6547–7434)	3157 (3124–3190)	2817 (2773–2861)	3485 (3436–3535)	3485 (3370–3603)	3323 (3211–3438)	3636 (3519–3756)
1–4	590 (546–636)	515 (458–577)	665 (600–736)	242 (237–246)	239 (233–246)	244 (238–250)	207 (180–237)	210 (183–240)	204 (177–234)
5–9	172 (152–195)	181 (152–215)	163 (135–195)	65 (63–67)	55 (53–58)	73 (70–77)	70 (55–88)	68 (53–86)	72 (56–91)
10–14	146 (129–166)	160 (134–189)	133 (109–161)	62 (60–64)	51 (49–54)	72 (69–75)	59 (45–76)	54 (41–70)	63 (48–81)
15–19	308 (279–339)	189 (158–225)	429 (380–482)	132 (129–135)	104 (100–108)	160 (155–164)	97 (79–118)	88 (71–108)	106 (87–128)
20–24	390 (355–428)	355 (310–405)	433 (378–494)	174 (170–178)	143 (138–148)	206 (200–212)	142 (120–167)	120 (99–143)	164 (140–191)
25–29	898 (839–960)	874 (795–957)	929 (841–1023)	226 (221–231)	217 (210–223)	236 (229–243)	167 (143–194)	139 (117–164)	195 (169–224)
30–34	1081 (1014–1151)	1240 (1142–1343)	902 (813–997)	326 (320–332)	321 (313–330)	330 (322–339)	214 (186–245)	172 (147–200)	257 (227–290)
35–39	1384 (1294–1479)	1597 (1456–1749)	1201 (1087–1325)	485 (477–494)	456 (444–467)	516 (504–528)	288 (256–323)	227 (198–259)	349 (313–388)
40–44	1621 (1512–1735)	1450 (1302–1611)	1774 (1620–1940)	695 (685–706)	621 (607–636)	770 (754–786)	391 (353–432)	313 (279–350)	470 (428–514)
45–49	1787 (1649–1934)	2283 (2057–2526)	1337 (1173–1517)	945 (931–959)	807 (789–827)	1081 (1059–1102)	537 (493–584)	413 (374–455)	661 (612–713)
50–54	2143 (1974–2324)	2248 (2010–2506)	2027 (1790–2287)	1240 (1221–1259)	997 (973–1022)	1478 (1448–1507)	828 (773–886)	687 (637–740)	969 (909–1032)
55–59	1810 (1648–1985)	1447 (1254–1661)	2264 (1994–2561)	1605 (1581–1628)	1217 (1189–1246)	1992 (1955–2030)	1219 (1152–1289)	968 (908–1031)	1478 (1404–1555)
60–64	2650 (2438–2876)	2479 (2206–2775)	2869 (2539–3229)	2238 (2207–2270)	1677 (1639–1715)	2817 (2766–2868)	1839 (1756–1925)	1533 (1457–1612)	2163 (2073–2256)
65–69	2825 (2575–3093)	2890 (2554–3258)	2756 (2388–3164)	3176 (3133–3220)	2445 (2393–2498)	3947 (3877–4017)	2735 (2633–2839)	2337 (2243–2434)	3162 (3053–3274)
70–74	6078 (5658–6521)	6084 (5521–6688)	6070 (5449–6743)	4834 (4774–4895)	3950 (3876–4024)	5847 (5750–5946)	4204 (4078–4333)	3713 (3595–3834)	4738 (4604–4875)
75–79	8044 (7377–8755)	7649 (6819–8551)	8656 (7564–9861)	7299 (7200–7399)	6335 (6214–6458)	8575 (8410–8743)	6337 (6182–6495)	5717 (5570–5867)	7057 (6893–7224)
80+	14,460 (13668–15,287)	15,984 (14933–17,090)	11,923 (10762–13,176)	14,084 (13970–14,199)	13,247 (13106–13,389)	15,620 (15427–15,815)	13,007 (12784–13,232)	12,263 (12047–12,482)	14,002 (13771–14,236)
Total	**1081 (1062–1100)**	**1108 (1081–1135)**	**1053 (1026–1080)**	**585 (583–587)**	**528 (525–530)**	**643 (640–646)**	**701 (650–755)**	**646 (597–698)**	**755 (702–811)**

*MR* mortality rate per 100,000 population, *LMIC* low- and middle-income countries

### Cause-specific mortality

**Fig. 2:**
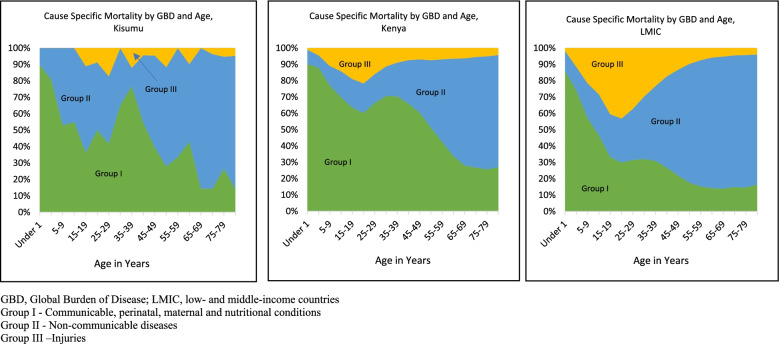
Cause-specific Mortality by GBD and Age, Kisumu, Kenya & LMICs

In Kisumu, we observed a high burden of Group I deaths for ages 0–44 years; 0–55 years in Kenya and 0–29 years in LMIC (Table 3 & Fig. 2). In general, deaths attributable to Group I diseases decreased with age, while those resulting from Group II increased with age.

**Table 3:** Cause-specific mortality per 100,000 population by GBD class and age, Kisumu, Kenya & LMIC 2019

	KISUMU				KENYA				LMIC			
Ages (Years)	Group I	Group II	Group III	All Cause	Group I	Group II	Group III	All Cause	Group I	Group II	Group III	All Cause
Under 1	4675	525	–	6058	2848	277	32	3157	3003	422	60	3485
1–4	326	78	–	589	213	18	11	242	153	30	24	207
5–9	195	173	–	172	50	8	7	65	40	15	15	70
10–14	105	86	–	146	43	10	9	62	27	15	17	59
15–19	147	212	45	307	84	23	25	132	32	25	39	97
20–24	153	125	27	390	105	31	38	174	43	38	61	142
25–29	447	439	184	898	150	40	36	226	53	52	62	167
30–34	850	450	–	1081	230	59	37	326	69	82	64	214
35–39	917	136	143	1385	342	100	43	485	89	132	67	288
40–44	732	554	59	1622	458	186	51	695	105	219	68	391
45–49	754	1071	87	1788	571	308	65	945	118	348	71	537
50–54	523	1135	219	2142	635	512	92	1240	146	602	80	828
55–59	686	1354	–	1810	681	813	111	1605	187	942	90	1219
60–64	1193	1307	269	2651	760	1333	145	2238	263	1470	106	1839
65–69	347	2054	–	2826	889	2086	201	3176	383	2211	141	2735
70–74	924	5310	235	6076	1298	3273	264	4834	626	3391	187	4204
75–79	2157	5543	426	8045	1872	5056	371	7299	929	5139	269	6337
80+	1978	11,207	659	14,460	3821	9661	602	14,084	2149	10,347	510	13,007
All	**516**	**512**	**56**	**1081**	**316**	**228**	**41**	**585**	**186**	**452**	**63**	**701**

*LMIC* low- and middle-income countries

Group I - Communicable, perinatal, maternal and nutritional conditions

Group II - Non-communicable diseases

Group III –Injuries

## Discussion

We found that the all-cause SMR in Kisumu County was one and a half times higher than that of all of Kenya and twice that of LMICs. The all-cause SMR in Kenya was 17% higher (659 vs. 547 per 100,000 population respectively) than that of all LMICs. In Kisumu County, death was caused by Group I diseases (communicable diseases, maternal conditions and nutritional deficiencies) and Group II (non-communicable diseases) diseases equally. Two verbal autopsy-based DSS studies in Western Kenya, however, found differing results. First, a study in a Kisumu County DSS site between 2011 and 2015 found that Group I diseases, accounted for 43% of all deaths with HIV/AIDS and malaria as the leading causes of death while non-communicable diseases represented 34% of deaths and injuries represented 7% [27]. Another DSS-based study in neighboring Siaya County between 2003 and 2010 ascribed 60% of deaths to communicable diseases and 37% of deaths to non-communicable diseases [8]. It has been thought that for Kenya in general communicable diseases continue to predominate the overall disease burden even as non-communicable diseases gradually increase with the aging population [28]. While this may be the case at the country level, our study findings indicate that Group II diseases are a notable cause of mortality in Kisumu County, which has higher mortality rates due to non-communicable diseases than Kenya as a whole. This finding needs to be explored further and considered during future public health programming in the county.

The 2015 and 2017 GBD studies found that Group I causes of death accounted for about one fifth of deaths globally [2, 29] and more than half of all deaths in low-income countries [29]. We found that Group I diseases represented 50.5 and 31.6% of all-cause mortality in Kenya and LMICs respectively. The patterns of all-cause mortality rates in Kenya are similar to hospital-based studies conducted in neighboring countries of Tanzania and Ethiopia, which reported more than half of their deaths were due to the Group I category while about one third were caused by Group II diseases [15, 16]. Overall, Group III causes (injuries) across all geographical areas of analysis had the lowest contribution to all-cause mortality.

Group II causes of death represented the highest burden of mortality among women in Kisumu while men experienced a higher burden of Group I related mortality. In Kenya, Group I diseases contributed to more than half of deaths among women (52.0%) and 49.2% of deaths among men. In LMICs, Group II diseases contributed more than half of the mortality overall (58.1%) and in both sexes. The proportion of adult deaths due to injuries in Kisumu was higher among men than women, similar to trends in other SSA countries [15, 30]. Across all geographical areas, there was a high burden of Group I causes of death in ages 0–44 with a switch to Group II CODs in older age groups. Infant mortality was notably high in all geographical areas but significantly higher in Kisumu County compared to Kenya and LMICs; high infant mortality rates in SSA have been documented [31, 32].

There are a few possible reasons why all-cause and cause-specific mortality rates in Kisumu differ from those in Kenya as a whole. First, Kisumu has an HIV prevalence more than three times that of the national prevalence, 17.5% compared to the national HIV prevalence of 4.9% [33]. While the country has made great gains in curbing the HIV epidemic, HIV remains one of the leading causes of mortality both in the country in general [28, 34] and in Kisumu in particular [27]. Second, Kisumu is in a region where malaria is highly endemic and a major cause of hospitalizations and deaths [27, 35, 36].

### Limitations

This study has several limitations. The two hospitals selected for this study were not randomly sampled from all hospitals within Kisumu County. The deaths in these hospitals represented 42% of the Kisumu East Registry, which captured 75% of all reported deaths in the county. Both hospitals are referral hospitals in an urban setting and are, thus, more likely to receive the most critical cases in the county and region. As such, patients attending these hospitals may be biased towards conditions that present with chronicity and complications, such as neonatal, pre-term and non-communicable conditions. This trend was observed in a hospital-based study in Tanzania [15]. While hospital mortality may not be a true reflection of deaths from various causes in the general population; it can give insight into the burden of diseases in the community and may be valuable in evaluating health care delivery systems of the country and, if followed serially, can be an important component of mortality surveillance. Slightly more than a third (34.8%; *n* = 296) of the eligible decedents who were brought in dead were excluded from the cause of death analysis as they had no accompanying hospital record or postmortem conducted. The potential bias for exclusion of these cases could not be determined. Additionally, there was evidence of poor record keeping or file storage, data incompleteness and unavailability of hospital files. Among the hospital deaths, 20% were excluded either because their files could not be retrieved, or cause of death could not be determined due to incomplete records. We assumed that the records obtained had accurate data and could not determine the potential bias of the missing records on cause-specific mortality. Kenya and LMICs mortality data are restricted to the multiple sources of data available at the Global Health Data Exchange with varying quality. Finally, we were unable to subtract deaths that occurred in Kisumu from overall Kenyan mortality data and deaths that occurred in Kenya from overall LMICs data. However, as Kisumu County represents only 1.4% of the population of Kenya, these corrections are unlikely to change our conclusions and, if we had been able to make them, would have made the differences only more pronounced.

## Conclusions

All-cause mortality is higher in Kisumu County than in Kenya as a whole. The county is faced with a high burden of both communicable and non-communicable diseases, and infant mortality remains of particular concern. It is important to consider the double burden of mortality due to both GBD Group I and Group II diseases, with special attention to management of conditions leading to infant mortality as well as HIV and malaria-related mortality, when prioritizing public health interventions. Our study has shown the importance of having reliable vital registry systems that collect complete and accurate data in all sub-areas of the country as the causes of deaths and trends in mortality can be heterogeneous. Such data will help the country to efficiently work towards attaining national targets as well as global sustainable development goals related to mortality.

## Data Availability

Data and materials used for this study are available from the corresponding author upon request.

## Abbreviations

ANACoD: Analyzing Mortality Levels and Causes-of-Death
CDC: Centers for Disease Control and Prevention
COD: Cause of Death
CRVS: Civil Registration and Vital Statistics
DSS: Demographic Surveillance Sites
GBD: Global Burden of Disease
HIV/AIDS: Human Immunodeficiency Virus Infection/Acquired Immunodeficiency Syndrome
IHME: Institute for Health Metrics and Evaluation (IHME),
ICD10: International Classification of Diseases and Related Health Problems 10th Revision
JOORTH: Jaramogi Oginga Odinga Teaching and Referral Hospital
KCRH: Kisumu County Referral Hospital
KEMRI: Kenya Medical Research Institute’s Scientific and Ethics Review Unit
LMICs: Low-and-middle income countries
NASCOP: National AIDS and STI Programme
SDI: Socio-demographic Index
SMR: Standardized Mortality Rate
SSA: Sub-Saharan Africa
UCSF: University of California, San Francisco
